# The Possible Relationship Between Sigmoid Dehiscence, Degree of Mastoid Pneumatization, and Sigmoid Sinus Position in Patients with Pulsatile Tinnitus

**DOI:** 10.3390/diagnostics16060914

**Published:** 2026-03-19

**Authors:** Burak Bilecenoğlu, Tuğçe Akın, Berin Tuğtağ Demir, Ömer Korkmazyürek, Ali Köksal, Kaan Orhan

**Affiliations:** 1 Department of Anatomy, Faculty of Medicine, Ankara Medipol University, Eti, Celal Bayar Blv. No:88/1, Ankara 06570, Turkey; tugce.akin@ankaramedipol.edu.tr; 2 Department of Clinical Anatomy, Institute of Health Sciences, Istanbul Medipol University, Istanbul 34820, Turkey; 3 Department of Otorhinolaryngology, Derik State Hospital, Kale Mahallesi, Diyarbakır Blv. No:70, Mardin 47800, Turkey; omerkorkmazyurek@gmail.com; 4 Department of Radiology, Bayındır Hospital Söğütözü, Kızılırmak Mahallesi, Söğütözü, 1443. Cd. No:17, Ankara 06250, Turkey; koksala72@gmail.com; 5 Department of Dentomaxillofacial Radiology, Faculty of Dentistry, Ankara University, Emniyet, Mevlana Blv. No:19/1, Ankara 06560, Turkey; call53@yahoo.com

**Keywords:** sigmoid sinus, pulsatile tinnitus, cone-beam CT, mastoid pneumatization

## Abstract

**Objective**: This study aimed to determine the relationship between sigmoid sinus dehiscence (SSD), sigmoid sinus topography, mastoid pneumatization, and adjacent temporal bone structures in patients with pulsatile tinnitus (PT). **Methods**: A retrospective analysis was performed on 344 temporal bone cone-beam computed tomography (CBCT) scans (172 PT patients and 172 age- and sex-matched controls). The degree of mastoid pneumatization, presence and size of SSD, sinus topography, and distances between the sigmoid sinus and key landmarks—the lateral semicircular canal (LSCC), jugular bulb (HJB), and external auditory canal (EAC)—were measured. Quantitative and qualitative characteristics were compared between groups, and independent predictors of PT were identified using multivariate logistic regression. **Results**: Compared to controls, SSD was substantially more common in the PT group (115/172 vs. 44/172, *p* < 0.001). Patients with PT had significantly larger anteroposterior and vertical sigmoid sinus dehiscence diameters (4.61 ± 0.99 mm vs. 3.87 ± 0.25 mm and 3.37 ± 0.47 mm vs. 2.92 ± 0.14 mm, respectively; both *p* < 0.01). Additionally, in the PT group, the sigmoid sinus was situated closer to the lateral semicircular canal, jugular bulb (JB), and external auditory canal (all *p* < 0.01). **Conclusions**: Venous pulsatile tinnitus was substantially correlated with sigmoid sinus dehiscence, sinus topography, and decreased sinus–EAC distance. Quantitative CBCT evaluation of these anatomical relationships could help with surgical planning and enhance diagnostic evaluation.

## 1. Introduction

Pulsatile tinnitus (PT) is a rhythmic auditory perception synchronized with the heartbeat, most commonly arising from venous pathologies such as sigmoid sinus wall anomalies, including dehiscence and diverticulum [1,2,3]. The intact bony sigmoid plate normally functions as an acoustic barrier that prevents the transmission of vascular noise to the cochlea; when disrupted, venous flow sounds can directly propagate, resulting in PT [3,4]. Venous PT has been explained by a number of pathophysiological models. Direct hydroacoustic transmission via a dehiscent sigmoid plate and vibration of the exposed sinus wall generating vibroacoustic energy are the two primary mechanisms. Similar anatomical findings in asymptomatic individuals, however, imply that these relationships are associative rather than strictly causal [4]. Although sigmoid sinus dehiscence (SSD) is frequently detected in PT patients, its presence in asymptomatic individuals indicates that other factors such as the extent of dehiscence, the sinus location, and the degree of mastoid pneumatization may influence symptom expression [1,2].

The mastoid air cell system can modulate sound propagation; extensive pneumatization may amplify resonance, whereas limited aeration could act as an acoustic barrier [2,5]. The spatial relationship between the sigmoid sinus and the mastoid bone varies considerably, influenced by pneumatization patterns, anatomical dominance, and developmental factors, all of which are critical for both etiological understanding and surgical safety [6]. Moreover, recent evidence suggests that sigmoid plate dehiscence may be an acquired condition related to chronic hemodynamic stress or early bone demineralization, such as osteoporosis [3].

Several studies have demonstrated that both structural abnormalities of the sigmoid plate and the spatial configuration of the sinus play a critical role in the generation of venous pulsatile tinnitus. Zhao et al. [7] and Dong et al. [1] showed that thinning or dehiscence of the sigmoid plate facilitates the transmission of low-frequency vascular pulsations toward the cochlea. Liu et al. [2] further demonstrated that the interaction between dehiscence and mastoid pneumatization modulates sound conduction within the temporal bone system. In addition, Geng et al. [8] reported that the spatial proximity between the sigmoid sinus and the external auditory canal affects the acoustic transfer pathway. These findings support the concept that SSD morphology, sinus topography, and the SSD–EAC distance contribute to the pathophysiological mechanisms underlying venous PT and justify their inclusion as key parameters in the present study.

Despite growing awareness of venous PT and its anatomical correlations, the interplay between SSD, mastoid pneumatization, and sinus topography has not been comprehensively evaluated. A number of classification schemes have been put forth in earlier research to characterize the topography of the sigmoid sinus and its clinical significance. While Sun et al. [9], Galal et al. [10], and Tsutsumi et al. [11] emphasized the variability of the EAC–sinus relationship and its implications for temporal bone and cochlear implant surgery, Nalbant et al. [12] and Demir et al. [13] showed that anterior or lateral displacement of the sinus can narrow the mastoid cavity and increase surgical complexity. While these studies emphasize the significance of sinus position, the majority rely on qualitative evaluations and do not incorporate adjacent temporal bone structures or dehiscence characteristics. Furthermore, none have investigated the role of these combined anatomical factors in venous pulsatile tinnitus. The current study offers a more thorough assessment of sinus morphology and elucidates its function in the pathophysiology of PT by integrating and quantitatively extending these previous classification techniques.

Therefore, the present study aims to investigate the relationship between the presence and characteristics of SSD, the mastoid pneumatization pattern, and the anatomical position of the sigmoid sinus to better elucidate their combined role in the pathophysiology of pulsatile tinnitus. We hypothesized that the presence or anatomical characteristics of sigmoid sinus dehiscence, mastoid pneumatization patterns, and sigmoid sinus topography would not show any significant association with the occurrence of pulsatile tinnitus.

## 2. Material and Methods

### 2.1. Study Material

A total of 344 CBCT scans of the temporal bone from patients aged 18–65 years were retrospectively evaluated. Patients with arterial, middle-ear, tumoral, paraganglioma-related, osseous, muscular, temporomandibular joint–related, or Eustachian tube–related causes of pulsatile tinnitus were excluded based on clinical evaluation, audiologic testing, and CTA/MRA when indicated. Only patients whose clinical and imaging characteristics were consistent with venous-type pulsatile tinnitus and without an alternative etiology were included. Only patients with persistent pulsatile tinnitus lasting ≥3 months were included. Patients with clinical findings suggestive of muscular myoclonus, temporomandibular joint dysfunction, or Eustachian tube–related symptoms were excluded. The images were obtained from the Radiology and Otorhinolaryngology archives of Bayındır Söğütözü Hospital, Ankara. Patients were divided into two cohorts: the pulsatile tinnitus (PT) group (*n* = 172) and the control group (*n* = 172). Within the past seven years, more than 450 individuals diagnosed with PT underwent computed tomography arteriography and venography (CTA + V) assessments at our institution. The following eligibility criteria were applied for study inclusion: (1) presence of unilateral, pulse-synchronous tinnitus that could be suppressed by ipsilateral jugular vein compression; (2) normal findings on laboratory testing, otoscopic, audiometric, and tympanometric examinations, as well as on digital subtraction angiography; (3) SSD identified as the primary etiological factor of PT, with alternative causes excluded; and (4) complete resolution of PT following surgical reconstruction, with no recurrence observed during follow-up. Ultimately, 172 participants (89 females and 83 males) met the inclusion criteria, including those diagnosed with PT. The mean age of all participants across both groups was 48.9 ± 2.56 years.

Unilateral pulsatile tinnitus was present in every patient in the PT group. As a result, the analysis only included the ear that was experiencing symptoms. To prevent measurement interdependence, the contralateral asymptomatic side was not examined. Only one ear per subject was included in the control group in order to preserve statistical independence and comparability. The side was matched to the PT group’s distribution of afflicted sides.

**Figure 1:**
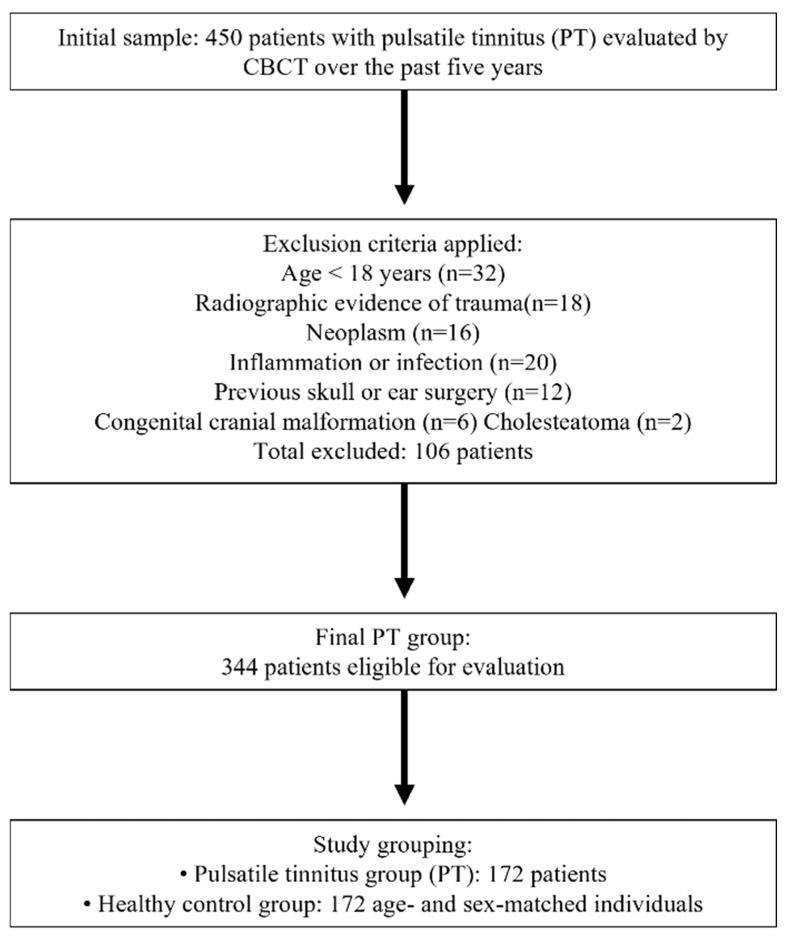
Flowchart of study design.

For comparison, 172 patients who underwent CTA + V imaging were recruited as the control group. Their inclusion criteria were as follows: matched for age and sex, and absence of PT or confirmed intracranial hypertension. The detailed selection flow of the study cohort is illustrated in Figure 1.

### 2.2. Imaging Protocol

All CBCT scans were acquired using the same imaging device (Planmeca Oy, Helsinki, Finland) with standardized parameters: 110 kVp, 5 mA, voxel size 0.25 mm^3^, slice thickness 0.3 mm, and a field of view of 18 × 16 cm.

Images were evaluated using RadiAnt DICOM Viewer (version 2025.2, 64-bit; Medixant, Poznań, Poland) (64-bit) software in multiplanar reconstruction (axial, coronal, and sagittal) modes under optimal brightness and contrast.

### 2.3. Image Evaluation and Parameters

Two observers, one anatomist and one radiologist, each with over 10 years of professional experience, independently evaluated all images. For both continuous and categorical variables, inter-observer reliability was evaluated independently. For categorical parameters (the presence and location of sigmoid sinus dehiscence and the classification of sinus topography), Cohen’s kappa coefficient was employed. The intraclass correlation coefficient (ICC), which is based on a two-way random-effects model with absolute agreement, was used to assess inter-observer agreement for continuous quantitative measurements, such as SSD diameters and all distance measurements (EAC–SSD, HJB–SSD, LSCC–SSD). ICC values were interpreted as follows: <0.50 poor reliability, 0.50–0.75 moderate reliability, 0.75–0.90 good reliability, and >0.90 excellent reliability.

**Figure 2:**
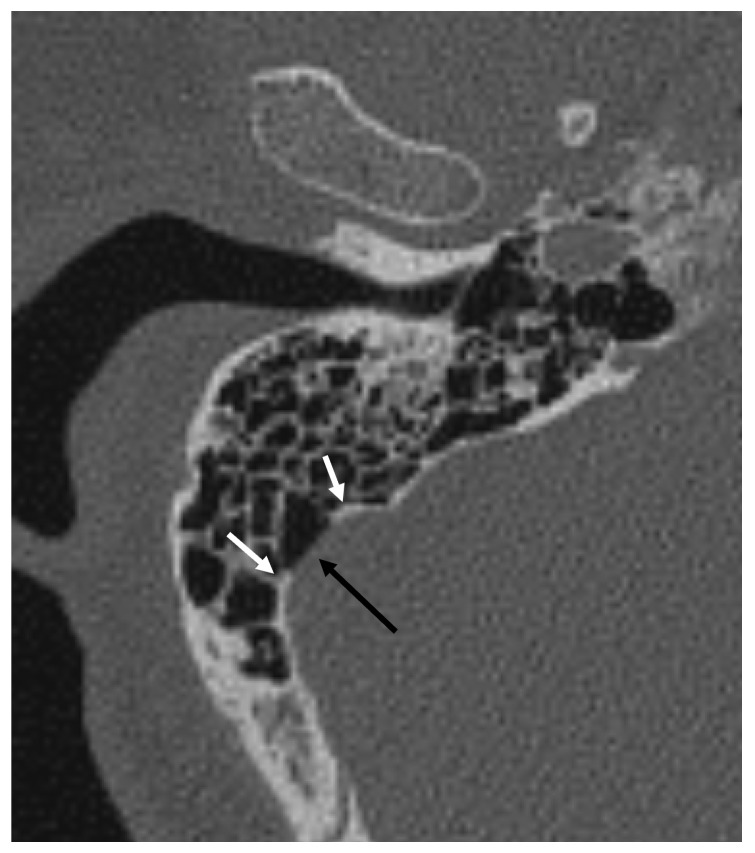
Axial CBCT image of the bony window shows a right dehiscent sigmoid sinus (black arrow). Inflection points of the sigmoid sulcus’s maximal curvature (white arrows) divide the wall into anterior, lateral and posterior ones.

**Figure 3:**
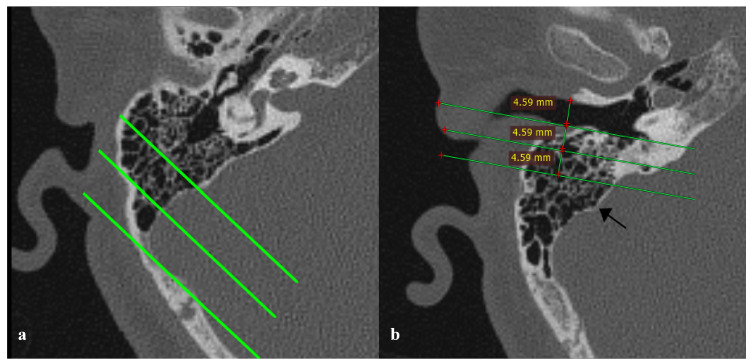
(**a**) Type 3, well-pneumatized temporal bone in which the mastoid air cells extended into the region between the middle and lateral lines; (**b**) Type 1 sigmoid sinus (SS), with the anterior margin of the SS (black arrow) positioned posterior to the third reference.

**Figure 4:**
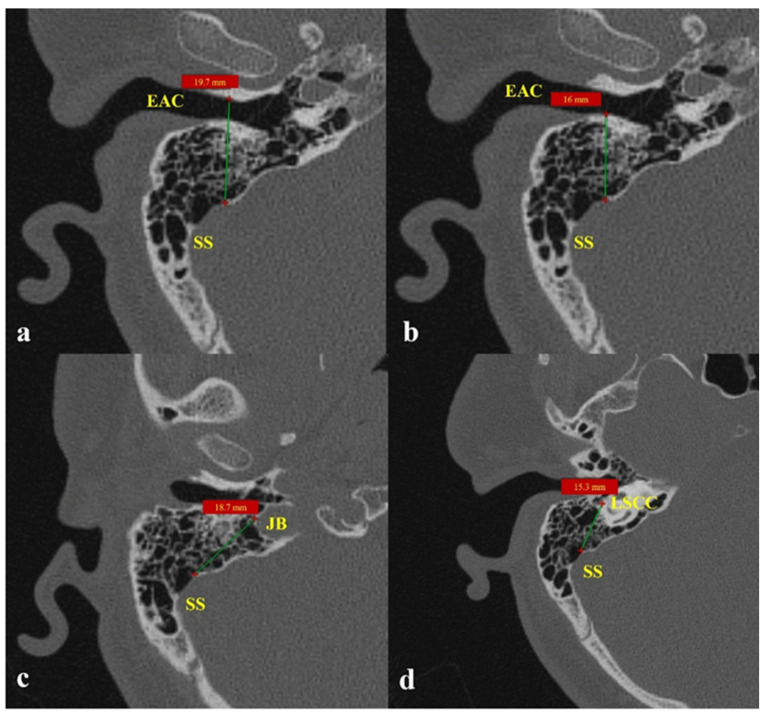
Distances from sigmoid sinus dehiscence (SSD) to adjacent key anatomical structures on axial CBCT images. (**a**) Shortest distance from SSD to the anterior wall of the external auditory canal (EAC); (**b**) Shortest distance from SSD to the posterior wall of the EAC; (**c**) Shortest distance from SSD to the jugular bulb (JB); (**d**) Shortest distance from SSD to the inferior border of the LSCC.

Each temporal bone was analyzed for the following parameters:

(1) Presence, location and diameter of SSD: The number, distribution, and extent of SSD on the symptomatic side were systematically evaluated using axial CBCT images. Vertically, the sigmoid plate was divided into upper, middle, and lower segments with reference to the common crus and cochlear fenestra. In the horizontal plane, it was partitioned into anterior, lateral, and posterior walls, defined by the anterior and posterior inflection points of the sigmoid sulcus (Figure 2). Diameters were measured on reconstructed axial slices, and the total SSD area was determined by summing the diameters across all sections [7].

(2) Presence of sigmoid plate air cell: Sigmoid plate classified as “wall pattern” (intact cortical bone adjacent) or “air cell pattern” (air cells abutting the dehiscent wall).
(3) Mastoid pneumatization pattern: Mastoid aeration was evaluated relative to the SS and the labyrinth, following previously described methods [2]. Temporal bone pneumatization was categorized into four types: hypopneumatized, moderately pneumatized, well pneumatized, and hyperpneumatized. Classification was performed on axial CBCT images at the level where the malleoincudal complex displayed an ice-cream-cone-shaped appearance. Three parallel lines oriented 45° anterolaterally were drawn through the most anterior, lateral, and posterior points, respectively, with reference to the posterior margin of the SS. (Figure 3a).
(4) Sigmoid sinus topography: The position of the sigmoid sinus was determined based on the classification systems proposed by Nalbant [8] and Demir [9] in their study. The classification was further validated with reference to the works of Galal et al. [10], Park et al. [11], Sun et al. [12], and Tsutsumi et al. [13]. On axial images, three parallel lines were drawn to determine SS position: the first line was drawn parallel to the posterior wall of the external auditory canal (EAC) at the level where the EAC appeared widest and longest; the second line was drawn posterior to the first, at a distance equal to the EAC width; and the third line was drawn parallel to the second, at the same interval as between the first and second lines. If the most anterior margin of the SS lay between the posterior wall of the EAC and the first line, it was classified as Type 3; if located between the first and second lines, it was classified as Type 2; and if positioned posterior to the third line, it was classified as Type 1 (Figure 3b).

(5) Distances from SSD to adjacent key structures EAC anterior wall, EAC posterior wall, jugular bulb, lateral semicircular canal (LSCC): Measurements were obtained on axial CBCT images at or near the level of the LSCC, where the surrounding ear structures are optimally visualized. The measurement points were standardized according to surgical relevance. In order to guarantee that the distances in this study directly correspond to anatomical landmarks that are frequently seen and accessed during otologic and neurotologic procedures, the measurement points were “standardized according to surgical relevance.” Mastoidectomy, posterior tympanotomy, and sigmoid sinus wall reconstruction frequently involve the anterior and posterior walls of the EAC, the dome of the jugular bulb, and the inferior border of the LSCC. Our morphometric data’s clinical applicability was improved by using these surgically significant and repeatable landmarks, which enabled the measurements to represent actual operative spatial relationships: (1) SSD–EAC anterior wall—from the anterior margin of the dehiscence to the anterior wall of the EAC; (2) SSD–EAC anterior wall—from the posterior margin of the dehiscence to the posterior wall of the EAC; (3) SSD–JB—from the anterior border of the dehiscence to the dome of the jugular bulb; and (4) SSD–LSCC—from the anterior margin of the dehiscence to the inferior border of the LSCC [6,14] (Figure 4).
(6) Associated vascular variations including high jugular bulb (HJB), emissary vein connection, and transverse sinus stenosis: A HJB was defined as a bulb located above the level of the internal auditory canal floor. The venous drainage system was considered co-dominant when the difference between the diameters of the bilateral mid-transverse sinuses was ≤3 mm; otherwise, the side with the larger diameter was regarded as dominant. A transverse sinus showing a focal luminal narrowing of ≥50% on axial images was defined as stenotic, while a mastoid emissary vein with a diameter exceeding 4.0 mm was considered abnormal. HJBs, mastoid emissary veins, transverse sinus dominance, and stenosis were all assessed on axial CBCT images. The height of the pituitary gland and fossa were separately measured on sagittal CBCT sections where the pituitary stalk was visualized [7].

### 2.4. Statistical Analyses

All statistical analyses were performed using IBM SPSS Statistics for Windows, Version 20.0 (IBM Corp., Armonk, NY, USA). Categorical variables were compared using the Chi-square test. Continuous variables were tested for normality using the Shapiro–Wilk test and analyzed with the independent-samples *t*-test. Correlations among continuous parameters were evaluated using Pearson correlation coefficients. Logistic regression analysis was applied to identify independent predictors of PT. A *p*-value less than 0.05 was considered statistically significant. Because multiple morphometric parameters were evaluated, each representing distinct anatomical relationships with independent clinical relevance, group comparisons were performed using independent-samples *t*-tests for each variable. To reduce the risk of type 1 error due to multiple comparisons, *p*-values were interpreted cautiously and confirmed using multivariate logistic regression to identify independent predictors of PT. A Bonferroni-adjusted significance threshold was additionally considered.

Receiver operating characteristic (ROC) curve analysis was performed to evaluate the diagnostic performance of significant morphometric parameters in discriminating PT patients from controls. The area under the curve (AUC) with 95% confidence intervals was calculated for each variable. Optimal cut-off values were determined using the Youden index (maximum sensitivity + specificity − 1). Sensitivity, specificity, and corresponding threshold values were reported. IBM SPSS Statistics version 20.0 (IBM Corp., Armonk, NY, USA) was used for all analyses.

## 3. Results

The study comprised a total of 344 temporal bone CBCT scans obtained from 172 patients diagnosed with PT and an equivalent number of healthy controls. Accordingly, 172 symptomatic ears in the PT group and 172 ears in the control group were included in the final analysis. The mean age of the subjects was 48.9 ± 2.56 years, with no statistically significant difference between the groups regarding age or sex distribution (*p* > 0.05). A post hoc power analysis indicated a statistical power of >0.90 for detecting the observed group difference in anterior EAC–SSD distance.

Baseline demographic and audiological characteristics of the PT and control groups are summarized in Table 1.

**Figure 5:**
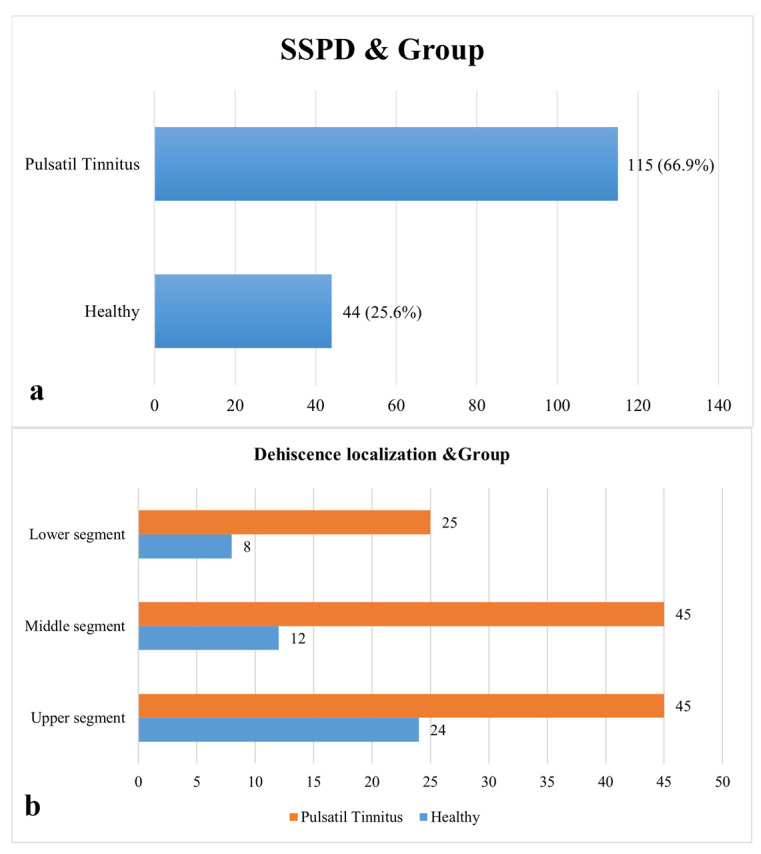
Presence and type of dehiscence by groups (SSPD: Presence of Sigmoid Sinus Dehiscence). (**a**) Prevalence of SSD/SSPD by Group; (**b**) Localization of Dehiscence and Group Distribution.

Age (PT: 48.7 ± 2.61 years; control: 49.2 ± 2.52 years; *p* > 0.05) and sex distribution (PT: 89 females, 83 males; control: 89 females, 83 males; *p* > 0.05) were matched between the PT and control groups. Both groups had normal air- and bone-conduction thresholds according to audiometric evaluation, and there were no statistically significant differences in mean pure-tone averages or air-bone gap values (all *p* > 0.05). In order to make group comparisons easier to understand, sigmoid sinus dehiscence (SSD), which was found in 115 patients in the PT group and 44 subjects in the control group, is now specifically re-ported (Figure 5).

With kappa values of 0.85 for SSD presence and location and 0.90 for sinus topography classification, the inter-observer agreement for categorical variables was excellent. ICC analysis showed exceptional reliability for continuous morphometric measurements: SSD diameter measurements (ICC range: 0.82–0.90), anterior EAC–SSD distance (ICC = 0.86), posterior EAC–SSD distance (ICC = 0.84), HJB–SSD distance (ICC = 0.89), and LSCC–SSD distance (ICC = 0.83).

Quantitative morphometric analysis demonstrated significant anatomical differences between the patient group (PT) and the control group (Table 2). A moderate positive correlation has been observed between the relevant parameters and the anteroposterior and vertical diameters of the SSD. As the dehisence diameter increases, there is a tendency for the correlation with EAC (r = 0.94, *p* = 0.014), LSCC (r = 0.089, *p* = 0.006), and HJB (r = 0.88, *p* = 0.023). The mean distance between the anterior wall of the EAC and the SSD was significantly greater in the patient group (30.13 ± 3.37 mm) than in the control group (29.23 ± 4.70 mm) (F = 14.62, *p* < 0.001). In addition, a significant reduction in the distances between the posterior EAC wall, jugular bulb, LSCC and the sigmoid sinus was observed in the PT group (*p* < 0.01 for all).

The mean anteroposterior and vertical diameters of the dehiscence were found to be greater in patients with PT compared to the control group (4.61 ± 0.99 mm vs. 3.87 ± 0.25 mm and 3.37 ± 0.47 mm vs. 2.92 ± 0.14 mm, respectively; *p* < 0.01). No statistically significant difference was observed between the groups with regard to the distance from the dehiscence to the outer mastoid cortex (*p* = 0.064) (Table 2).

**Figure 6:**
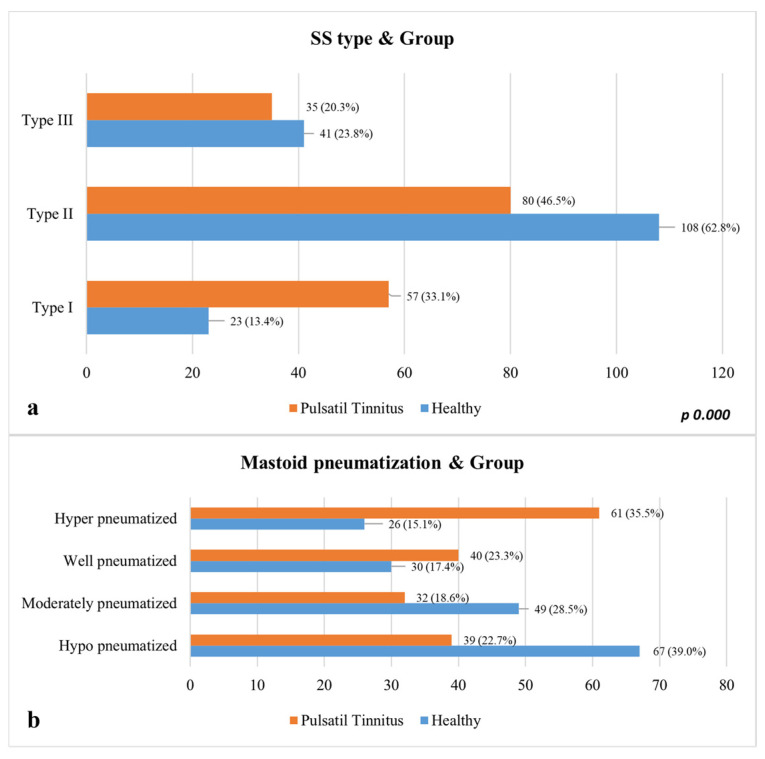
Distribution of mastoid pneumatization and sigmoid sinus (SS) types by group. (**a**) Distribution of Sigmoid Sinus (SS) Types by Group; (**b**) Comparison of Mastoid Pneumatization Degrees.

The qualitative assessment revealed the presence of air cells adjacent to the sigmoid plate in 38.4% of the control group and 52.9% of the patient group (*p* = 0.023). Despite the absence of statistical significance in the prevalence of HJB (*p* = 0.547), vascular variations, including emissary vein connection (7.6% vs. 18%), bilateral transverse sinus stenosis (12.8% vs. 37.2%), and unilateral stenosis (ipsilateral = 37.2%, contralateral = 49.4%), were found to be significantly more prevalent in the PT group (all *p* < 0.001) (see Table 3 and Figure 5 and Figure 6).

Sigmoid plate dehiscence, lateral location of the sigmoid sinus, and EAC–sigmoid sinus distance has been identified as independent anatomical determinants for pulsatile tinnitus [1,2,7,8]. ROC analysis identified a cut-off value of 20.33 mm for the distance between the anterior EAC wall and the SSD, 9.45 mm for the distance between the posterior EAC wall and the SSD, and 13.78 mm for the HJB–SSD distance. In addition, for the posterior EAC wall, a 1 mm decrease in the EAC–SSD distance was associated with an increased likelihood of pulsatile tinnitus. The model’s explanatory power is moderate (Nagelkerke R^2^ = 0.155), and these three anatomical factors support the morphological basis of PT. This modest explanatory value suggests that non-anatomical factors also con-tribute to PT. Hemodynamic variables—such as venous flow velocity, turbulence, pressure gradients in the transverse–sigmoid system, and jugular outflow resistance—may amplify vascular pulsations independent of structural morphology. Intracranial pressure-related changes may likewise influence sinus wall vibration. Future studies combining anatomical imaging with hemodynamic assessment are needed to delineate how these dynamic factors interact with anatomical variations in producing PT.

Multivariate logistic regression analysis identified three independent anatomical predictors of pulsatile tinnitus: the presence of sigmoid plate dehiscence (odds ratio [OR] = 2952.87; 95% confidence interval [CI] = 1.87–4.41; *p* < 0.001), lateral (type 1) sigmoid sinus configuration (OR = 2.32; 95% CI = 1.16–4.65; *p* = 0.017), and reduced EAC–sinus distance 297 (OR = 1.071; 95% CI = 1.028–1.115; *p* = 0.001). Mastoid pneumatization demonstrated no statistically significant correlation with the occurrence of PT (*p* = 0.656) (Table 4).

## 4. Discussion

This study rejected the null hypothesis, as significant anatomical differences were identified between individuals with PT and healthy controls—particularly in the prevalence and dimensions of SSD, the lateral configuration of the sinus, and the reduced distance between the sinus and the EAC. Consistent with previous reports by Zhao et al. [7] and Liu et al. [3], our results support the concept that morphological variations of the sigmoid plate and adjacent temporal bone structures play a central role in the development of venous PT.

The mechanism of PT remains incompletely understood, yet accumulating evidence suggests a close relationship with abnormal venous hemodynamics [15,16,17,18,19,20]. Similar to Zhao et al., we observed that SSD, transverse sinus stenosis, and ipsilateral venous dominance were more common in PT patients than in controls, whereas no significant differences were found in the presence of a HJB or emissary vein connection. Importantly, our study provides a quantitative assessment, demonstrating that both the anteroposterior and vertical diameters of the dehiscence were significantly larger in PT patients. This finding indicates that not only the presence but also the extent of cortical bone loss contributes to symptom generation, potentially by enhancing vascular sound transmission through the temporal bone. Quantitative measurement of SSD on imaging may therefore help identify which lesions are more likely to produce clinical symptoms and guide surgical decision-making. Carefully measuring the size, quantity, and location of dehiscences in patients undergoing surgery can help choose the best technique and material for closing the dehiscence. The long-term surgical results of sigmoid sinus wall reconstruction in patients with PT were monitored in a study by Dai et al. [21] and it was noted that some patients experienced a recurrence of pulsatile tinnitus. In these situations, the likelihood of a successful surgical outcome has been found to be increased by careful surgical preparation combined with quantitative measurements of the dehiscence.

The sigmoid plate serves as a natural barrier preventing vascular pulsations from reaching the cochlea. When this barrier is thinned or dehiscent—often because of long-term hemodynamic stress on the anterior or lateral sinus wall—venous pulsations may be transmitted through air cells to the middle and inner ear [22,23]. Mastoid pneumatization degree modulates this transmission: smaller, hypo-pneumatized bones tend to dampen sound [24], whereas well-pneumatized structures can amplify or sustain it. Liu et al. experimentally demonstrated that temporal bone air cells influence sound propagation in SSD-induced PT, showing that low-frequency sounds are transmitted more efficiently, which explains why venous PT often presents as a low-pitched hum. In line with this acoustic model, our CBCT-based analysis revealed significantly shorter distances between the sigmoid sinus and the posterior wall of the EAC, HJB and LSCC in PT patients. These reduced separations likely facilitate the conduction of low-frequency pulsations toward the cochlea, reinforcing the hypothesis that spatial proximity and bone configuration jointly determine symptom expression. Additionally, because of their close proximity to the defect areas, these anatomical structures are more prone to complications in surgical procedures that target patient groups with short distances between the sigmoid sinus and the posterior wall of the EAC, HJB, and LSCC. Preoperative assessments can identify these connections and lower the risk of complications.

Comparable anatomical trends were also described by Hajare et al. [6], who reported that reduced sigmoid sinus–EAC distances were associated with advanced temporal bone pathology and hypo-pneumatized mastoids. Our findings mirror this pattern: the posterior EAC–sinus and HJB–sinus distances were significantly shorter in PT patients, whereas the anterior EAC–sinus distance showed minimal change. This consistency supports the notion that closer spatial relationships between vascular and osseous structures may predispose individuals to venous PT. Moreover, the results demonstrate the utility of CBCT in detecting subtle yet clinically meaningful morphologic variations of the temporal bone with lower radiation exposure compared to HRCT.

Identifying anatomical parameters that mediate sound conduction from the venous system to the cochlea is crucial for the diagnosis and management of PT. Dong et al. [1] reported that PT patients had significantly larger transverse and vertical SSD diameters than non-PT individuals, suggesting that larger dehiscences are more effective conduits for vascular sound. Our findings extend this evidence by showing that PT is not solely determined by dehiscence size, but also by sinus topography and spatial proximity to otologic landmarks. These parameters together form a structural “acoustic pathway” through which venous pulsations may be perceived as sound.

Although Dong et al. highlighted the relationship between dehiscence extent and PT symptoms, their imaging focus precluded evaluation of adjacent bone morphology. By including mastoid pneumatization and sinus orientation, our study provides a more comprehensive assessment of temporal bone architecture. The lack of significant differences in pneumatization volume between groups suggests that the conductive mechanism is driven primarily by the geometry and openness of the sinus wall rather than aeration volume alone. Clinically, these findings emphasize the importance of quantitative imaging in preoperative evaluation—specifically, measuring dehiscence dimensions and sinus–EAC distances can help determine which patients may benefit from sigmoid plate resurfacing or venous reconstruction. Restoration of the bony barrier may effectively eliminate the conductive pathway, as demonstrated in prior surgical reports.

This study is limited by its retrospective design and imaging-based analysis, which prevents causal inference regarding the relationship between bone dehiscence and PT perception. Although CBCT provides excellent spatial resolution, its capacity to assess hemodynamic flow dynamics is restricted. Future research combining quantitative imaging with computational flow models or intraoperative verification could clarify how venous pressure and flow velocity contribute to SSD formation and symptom intensity. Expanding sample size and including longitudinal follow-up would also help determine whether SSD progression correlates with clinical changes over time. Additionally, the Tinnitus Handicap Inventory (THI) and other standardized questionnaires intended to gauge the severity of tinnitus or its effect on patients’ quality of life were not used in this study. This restriction makes it impossible to determine a direct relationship between the severity of the patients’ symptoms and the size of the dehiscence.

## 5. Conclusions

A number of variables, such as sinus shape, proximity to the EAC, and SSD, greatly impact the development of venous PT. The current findings show that spatial geometry and the cortical integrity of the sinus wall are the primary determinants of sound transmission, despite the secondary influence of mastoid pneumatization. Clinical professionals can find patients who might benefit from surgical reconstruction or other focused management techniques by using quantitative CBCT-based evaluation of these parameters, which also offers useful diagnostic information. The management of venous pulsatile tinnitus may benefit from preoperative planning that includes a thorough assessment of sigmoid sinus morphology and its relationship to nearby temporal bone structures. This could lower diagnostic uncertainty and enhance results.

## Figures and Tables

**Table 1:** Baseline demographic and clinical characteristics. Data are presented as *n* (%) or mean ± standard deviation. Categorical variables were compared using the chi-square test and continuous variables using the independent-samples *t*-test.

	PT (*n* = 172)	Control (*n* = 172)	*p*
Smoking status, n (%)	45 (26.2%)	56 (32.5%)	0.847
Hypertension, n (%)	12 (7%)	10 (6%)	0.945
Diabetes, n (%)	26 (15.1%)	24 (14%)	0.755
Migraine, n (%)	19 (11%)	12 (7%)	0.870
Body mass index (BMI) (kg/m^2^), mean ± SD	25.89 ± 3.45	23.87 ± 3.12	0.950

**Table 2:** Comparison of quantitative anatomical measurements between the pulsatile tinnitus (PT) and control groups. Independent samples *t*-test results showing mean distances and diameters related to the sigmoid sinus dehiscence (SSD). Statistically significant differences are highlighted in bold (*p* < 0.05) (EAC: External auditory canal; HJB: High jugular bulb; LSCC: Lateral semicircular canal); *t:* Independent samples *t*-test value.

Parameters	Group		*t*	%95 CI	*p*
	PT	Health		Lower-Upper	
Shortest distance between anterior wall of EAC and SSD	30.13 ± 3.37	29.23 ± 4.70	−2.89	−1.51, 0.29	0.000
Shortest distance between the posterior wall of the EAC and the SSD	11.76 ± 3.25	14.30 ± 4.21	−1.87	−1.10, 0.02	0.006
Shortest distance between the HJB and SSD	16.38 ± 6.38	20.31 ± 8.75	−3.78	2.78, 5.08	0.005
Shortest distance from the LSCC with SSD	12.56 ± 4.25	16.20 ± 6.02	−6.20	2.86, 4.42	0.002
Distance between the dehiscence, mastoid cortex and the outer bone wall	7.19 ± 383	8.24 ± 3.95	9.12	0.089, 1.97	0.064
Dehiscence anteroposterior diameter	4.61 ± 0.99	3.87 ± 0.25	11.60	1.80, 3.41	0.002
Dehiscence vertical diameter	3.37 ± 0.47	2.92 ± 0.14	10.36	1.06, 2.63	0.000

**Table 3:** Comparison of qualitative anatomical and vascular variations between the PT and control groups. Chi-square test results for the presence of sigmoid plate air cells, high jugular bulb, emissary vein connection, and transverse sinus stenosis (bilateral, ipsilateral, and contralateral). Superscripts ‘a’ and ‘b’ indicate group differences based on post hoc pairwise comparisons.

	Group		*p*
	Healthy (*n* = 172)	PT (*n* = 172)	
Presence of sigmoid plate air cell	66 ^a^ (38.4%)	91 ^b^ (52.9%)	0.023
High jugular bulb	40 ^a^ (23.3%)	40 ^a^ (23.3%)	0.547
Emissary vein connection	13 ^a^ (7.6%)	31 ^b^ (18%)	0.007
Bilateral transverse sinus stenosis	22 ^a^ (12.8%)	64 ^b^ (37.2%)	0.000
Ipsilateral transverse sinus stenosis	29 ^a^ (16.9%)	64 ^b^ (37.2%)	0.000
Contralateral transverse sinus stenosis	33 ^a^ (19.2%)	85 ^b^ (49.4%)	0.000

**Table 4:** Multivariate logistic regression analysis identifying independent anatomical predictors of pulsatile tinnitus. Variables included sigmoid plate dehiscence, sigmoid sinus type, mastoid pneumatization, and external auditory canal (EAC)–sinus distance. Odds ratios (Exp(B)) with 95% confidence intervals (CI) are presented.

Variable	B	*p*	OR (Exp(B))	95% GA (Upper-Lower)
Sigmoid plate defect/dehiscence (present)	1.054	<0.001	2.87	1.87–4.41
Sigmoid sinus type (1)	0.843	0.017	2.32	1.16–4.65
Sigmoid sinus type (2)	0.051	0.834	1.05	0.65–1.69
Mastoid pneumatization	0.045	0.656	1.05	0.86–1.28
Shortest distance between the posterior wall of the EAC and the SSD (mm)	0.069	0.001	1.071	1.028–1.115
Constant	−2.584	<0.001	—	—

## Data Availability

The data that support the findings of this study are available on request from the corresponding author. The data are not publicly available due to privacy or ethical restrictions.
